# Implementation of Pediatric Flexible-Endoscopic Evaluation of Swallowing: A Systematic Review and Recommendations for Future Research

**DOI:** 10.1007/s00455-022-10446-0

**Published:** 2022-04-17

**Authors:** Jana Zang, Saskia Kiehn, Till Flügel, Jana-Christiane Koseki, Almut Nießen, Susan Hyoungeun Kim, Christina Pflug, Julie Cläre Nienstedt

**Affiliations:** Department of Voice, Speech and Hearing Disorders, Center for Clinical Neurosciences, University Medical Center Hamburg‐Eppendorf, Martinistrasse 52, 20246 Hamburg, Germany

**Keywords:** Pediatric FEES protocol, Deglutition disorder, Pediatric swallowing disorders, Breastfeeding, Bottle-feeding

## Abstract

**Background:**

Although pediatric flexible-endoscopic evaluation of swallowing (FEES) has developed into a standard in dysphagia diagnostics, there are no valid protocols and procedures for children available to date.

**Objective:**

This systematic PROSPERO-registered review aimed to identify implementation protocols for pediatric FEES described in research studies, and to analyze them in detail concerning procedural steps, equipment, and reported outcome.

**Methods:**

Included were all studies reporting a pediatric FEES protocol for children aged 0–18 years, if they described at least two criteria defined in advance. The databases MEDLINE and CINHAL were searched systematically from January 2000 to February 2021. Risk of bias for included studies was assessed using the National Institutes of Health (NIH) quality assessment tool for observational cohort and cross-sectional studies. A narrative synthesis of the FEES protocols was conducted and the results compared in tabular form.

**Results:**

In total 22 studies were included, reporting on FEES in 1547 infants, children, and adolescents with a wide range of diagnoses. It was possible to identify protocols related to all age groups in general as well as to particular groups such as breastfed or bottle-fed infants. None of the included studies demonstrated a good methodological quality; all studies had missing data. Uniform implementation for sub-groups could not be determined. The reported outcome of FEES examinations could not be compared.

**Discussion:**

None of the included studies showed good methodological quality and a significant amount of data were missing; the review still offers a systematic basis for future research to close the serious gap in the area of pediatric FEES. A proposal is made for a minimum requirement for pediatric FEES protocols in scientific studies.

## Introduction

Flexible-endoscopic evaluation of swallowing (FEES) is a feasible and safe instrumental swallowing assessment procedure in children of all ages [1]. Langmore [2] recently published a historical review of FEES, highlighting the increasing use in children. The benefits are: the identification of anatomical abnormalities, the ability to assess the exact diet with food and liquids rather than barium in the child's preferred position, and the opportunity to examine while breastfeeding [2, 3]. Miller et al. [3] and Miller and Willging [1] recently published detailed protocols for carrying out pediatric FEES. These contain the classic FEES procedure according to Langmore [4] and a broad description of the types of swallowing modifications including compensatory strategies that can be utilized during FEES. Recommendations for the procedures in specific populations are given, however, valid scales for uniform evaluation are missing.

A recent systematic review [5] stated that FEES protocols for the adult population, especially for patients with a neurogenic main emphasis, are very well developed and well researched. Yet, even those protocols contain disagreements and inaccuracies. A systematic review of quantitative instrumental swallowing assessment in children [6] was unable to include a single study of pediatric FEES from the past 20 years due to methodological weaknesses in the available studies.

Based on these shortcomings, the aim of this review was to (i) summarize the implementation protocols for pediatric FEES described in research studies and (ii) analyze the protocols in detail with regard to procedural steps, equipment, and reported outcomes.

The primary research questions are “*What implementation protocols for pediatric FEES are described in scientific studies, including technical and other equipment, and bolus texture and coloring?”* and “*What FEES-based outcomes concerning swallowing pathologies are reported and which scales are used to ensure objective classification? “* The secondary research question is “*Are implementation protocols able to be identified for certain sub-groups and what factors are detectable that make up these subgroup protocols?”.*

## Methods

### Search Strategy and Quality Assessment

This systematic review was registered on PROSPERO (CRD42021247396) and carried out roughly based on the “Preferred Reporting Items for Systematic Reviews and Meta-analyses Protocol” (PRISMA-P [7]). The MEDLINE and CINHAL databases were searched systematically from January 2000 to February 2021 using medical subject headings (MeSH) and keywords (Table 1). All eligible abstracts were screened for inclusion and exclusion criteria (Table 2). A manual search in the reference lists of the included articles was carried out to identify additional studies. Two reviewers (JZ and SK) independently evaluated the full texts for eligibility. An agreement was reached through discussion.

**Table 1:** Literature search strategies

Database	Search terms	Limitations	Results
MEDLINE	(child* OR infant OR („child" [MESH]) OR („infant" [MESH])) AND ("fiberoptic endoscopic evaluation of swallowing" OR „flexible-endoscopic evaluation of swallowing “ OR „endoscopic assessment “ OR „endoscopic evaluation “)) AND (dysphagia OR swallow* OR „swallowing disorder" OR „swallowing dysfunction" OR „deglutition disorder" OR "feeding disorder" OR ("deglutition disorder [MESH]))	Human 2000–2021 English	113
CINAHL	(children OR infant OR pediatric) AND ("fiberoptic endoscopic evaluation of swallowing" OR „flexible-endoscopic evaluation of swallowing “ OR „endoscopic assessment “ OR „endoscopic evaluation “) AND (dysphagia OR „swallowing disorder" OR „deglutition disorder")	Human 2000–2021 English	31 (2 in addition to MEDLINE)

**Table 2:** Inclusion and exclusion criteria for selecting abstracts and full texts

Inclusion criteria	Exclusion criteria
Children 0–18 years. with suspected dysphagia	Adults or mixed sample with less than 90% < 18 years
All diagnoses	Foreign body aspiration
Original work Description of a FEES protocol with at least two of the following criteria: Diameter and/or type of endoscope Positioning of the child Anesthesia and/or nasal decongestion Food coloring	Reviews No description of a pediatric FEES protocol

### Inclusion and Exclusion Criteria

All original scientific journal articles published in English that reported on a FEES protocol for the detection of dysphagia in children and described at least two of the predefined criteria for accurate performance were included (Table 2). There were no restrictions on study design.

### Risk of Bias and Quality Assessment

The risk of bias for each study was assessed with the National Institutes of Health (NIH) quality assessment tool for observational cohort and cross-sectional studies [8] (Table 3). One reviewer (JZ) carried out the assessment and one checked the results (SK). Disagreement was solved by discussion.

**Table 3:** NIH quality rating

Study	Q1	Q2	Q3	Q4	Q5	Q6	Q7	Q8	Q9	Q10	Q11	Q12	Q13	Q14	Quality
Armstrong et al. [12]	Y	N	Y	Y	N	NA	N	NA	NA	NA	N	NA	NA	N	Fair
Averin et al. [13]	Y	Y	Y	Y	N	NA	N	NA	Y	N	N	NR	NA	N	Fair
Beer et al. [27]	Y	Y	Y	Y	N	N	N	N	N	N	N	N	NA	N	Poor
Da Silva et al. [21]	Y	Y	NR	Y	N	N	N	NA	NA	NA	N	NR	NA	N	Fair
Hartnick et al. [28]	Y	N	NR	Y	N	N	N	NA	NA	NA	N	NR	NR	N	Poor
Kamity et al. [14]	Y	Y	NR	Y	N	N	N	NA	NA	NA	N	NA	NA	N	Fair
Leal et al. [22]	Y	Y	NR	Y	N	Y	N	NA	Y	N	Y	NR	NA	NA	Fair
Leder et al. [23]	N	Y	NR	NR	N	N	N	N	Y	N	N	NA	NA	NA	Poor
Leder & Karas [10]	Y	Y	N	N	N	N	NA	NA	NA	N	NA	NA	NA	NA	Poor
Link et al. [11]	Y	Y	Y	Y	N	N	N	Y	Y	N	N	N	NA	N	Fair
Marques et al. [15]	Y	Y	NR	Y	N	N	Y	NA	Y	NA	N	N	N	N	Fair
Mills et al. [16]	Y	Y	Y	Y	N	N	N	NA	Y	N	N	N	NA	N	Fair
Pavithran et al. [24]	Y	Y	NR	Y	N	N	N	NA	NA	NA	N	NA	NA	N	Fair
Richter et al. [25]	Y	Y	Y	Y	N	NA	CD	NA	Y	NA	N	NR	NR	N	Fair
Sitton et al. [29]	Y	Y	Y	Y	N	Y	NA	Y	N	CD	N	NR	Y	N	Fair
Suiter et al. [30]	Y	Y	NR	NR	N	N	N	Y	Y	N	N	N	NA	N	Poor
Suskind et al. [17]	Y	Y	NR	N	N	N	N	N	N	NR	N	NR	NR	N	Poor
Suterwala et al. [18]	Y	Y	NR	Y	N	N	N	NA	NA	NA	N	Y	Y	N	Fair
Ulualp et al. [31]	Y	Y	Y	Y	N	N	N	Y	Y	N	N	NR	NA	N	Fair
Umay et al. [32]	Y	Y	NR	Y	Y	N	N	Y	Y	Y	N	Y	Y	N	Fair
Vetter-Laracy et al. [19]	Y	Y	Y	Y	N	N	N	Y	Y	NA	N	NR	Y	N	Fair
Willette et al. [20]	Y	N	Y	Y	N	N	N	NA	NA	NA	N	N	NA	N	Fair

Q1: Was the research question or objective in this paper clearly stated? Q2: Was the study population clearly specified and defined? Q3: Was the participation rate of eligible persons at least 50%? Q4: Were all the subjects selected or recruited from the same or similar populations (including the same time period)? Were inclusion and exclusion criteria for being in the study prespecified and applied uniformly to all participants?; Q5: Was a sample size justification, power description, or variance and effect estimates provided?; Q6: For the analyses in this paper, were the exposure(s) of interest measured prior to the outcome(s) being measured?; Q7: Was the timeframe sufficient so that one could reasonably expect to see an association between exposure and outcome if it existed?; Q8: For exposures that can vary in amount or level, did the study examine different levels of the exposure as related to the outcome (e.g., categories of exposure, or exposure measured as continuous variable)?; Q9: Were the exposure measures (independent variables) clearly defined, valid, reliable, and implemented consistently across all study participants?; Q10: Was the exposure(s) assessed more than once over time?; Q11: Were the outcome measures (dependent variables) clearly defined, valid, reliable, and implemented consistently across all study participants?; Q12: Were the outcome assessors blinded to the exposure status of participants?; Q13: Was loss to follow-up after baseline 20% or less?; Q14: Were key potential confounding variables measured and adjusted statistically for their impact on the relationship between exposure(s) and outcome(s)?; Y: yes; N: no; NA: not applicable; NR: not reported; CD: cannot determine. Source: The National Institutes of Health (NIH) quality assessment tool for observational cohort and cross-sectional studies https://www.nhlbi.nih.gov/health-topics/study-quality-assessment-tools

### Data Synthesis

A narrative synthesis of the FEES protocols from the included studies was prepared roughly based on the SWiM (synthesis without meta-analysis) reporting guideline [9]. One reviewer extracted data from the studies (JZ) and three reviewers (TF, J-CK, SHK) checked the extracted data.

The modifiers (i) sample, (ii) FEES implementation, (iii) FEES equipment, and (iv) FEES outcome were transferred into tables. To ensure comparability of the data, they were standardized as much as possible. Children's ages were converted to months, and missing means and standard deviations were calculated where data from the studies allowed. The FEES procedural steps reported in the study protocols were summarized in standard terms based on Langmore [2] and Miller et al. [3] as follows: (1) *Observation of anatomical structures* with (a) *secretion management* and (b) *sensory testing,* (2) *direct assessment of swallowing*, (3) *compensatory strategies*, and (4) *sensory testing* (if tested at this point).

## Results

### Search Results

**Fig. 1:**
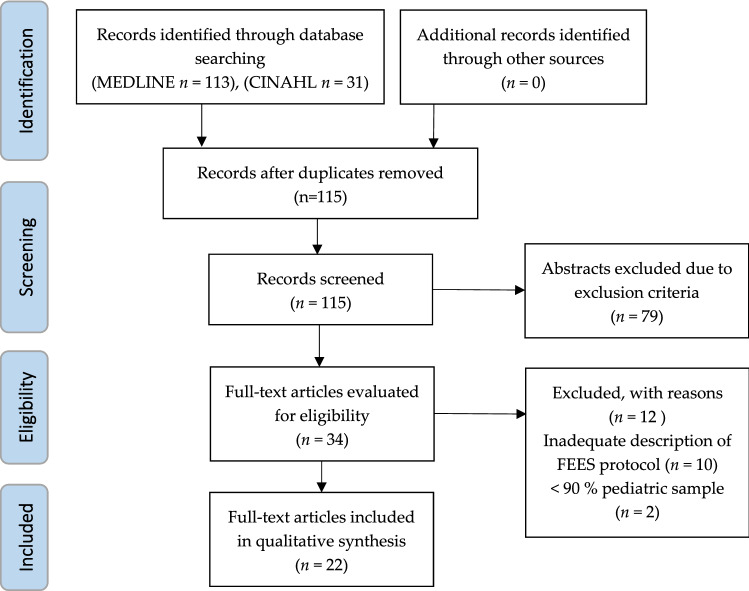
PRISMA flow diagram of the reviewing process [42]

A total of 115 records were identified through database search. After screening for inclusion and exclusion criteria, 22 full texts were included in the analysis (Fig. 1).

### Risk of Bias and Quality Assessment

All included studies were retrospective or prospective cross-sectional studies, including pilot studies and case series. No study achieved a good rating using the NIH quality assessment tool. Sixteen studies were rated *fair* and six *poor* (Table 3). Overall, substantial bias is to be expected because of the lack of methodological quality of the studies and a high number of missing parameters. Particularly critical was the description and definition of valid outcome parameters, especially considering the use of sound statistical methods such as justification of sample sizes and confounding variables.

### Study Population

The 22 included studies reported on 1547 children aged 0–18 years in total. The sample size per study ranged from five to 568 children. Two samples included a small number of young adults up to 20 [10] and up to 24 years [11]. The average age of the entire population could not be calculated due to missing values in some of the studies.

Nine studies exclusively focused on infants under 1 year of age [12–20]; five samples consisted of children younger than 12 months up to 3 years [21–25]; one study focused on children between 4 and 8 years [26], and a wide age distribution including infants, children and adolescents could be found in seven studies [10, 11, 27–30] (Table 4).

**Table 4:** Samples of the included studies

Study	N	Gender Sample^a^		Age in months^b^ (range; *Mdn/M* ± *SD*)^c^										
		(% f) Years:		0	1	2	3	4	5	6	7	8	9	10 +
Armstrong et al. [12]	5	40	Premature born, NICU (100%)	8.7–9.6 (PMA); *M* = 9.1 ± 0.3										
Averin et al. [13] Beer et al. [27]	63	NR	Hypoplastic heart syndrome, after Norwood operation	*Mdn* = 0.2										
da Silva et al. [21]	30	47	Traumatic brain injury (4), cerebral palsy (6), encephalopathy (5), encephalitis (3), genetic syndrome (4), multiple congenital anomalies (3), complex cerebellar malformation (1), leukodystrophy (1), epilepsy (2), progressive dystonia (1)	10–216; *Mdn* = 60										
Hartnick et al. [28]	30	40	Cerebral palsy (11), genetic malformations (4), gastroesophageal reflux (4), respiratory diseases (18), other (6)	10.5–37.3; *M* = 25.8 ± 21.2										
Kamity et al. [14]	568	NR	Structural (35%), neurologic (33%), pulmonary (6%), genetic (7%), gastrointestinal (10%), cardiovascular (3%), metabolic (1%), prematurity (4%), psychiatric (1%)	2–204; *Mdn* = 30										
Leal et al. [22]	5	20	Premature born, bronchopulmonary dysplasia, NICU (100%)	8.4–9.6 (CGA); *M* = 8.97 ± 0.4										
Leder et al. [23]	9	56	Congenital zika syndrome (100%)	9.5–16.8; *M* = 9.8										
Leder & Karas [10]	5	40	Mechanically ventilated via tracheotomy: Bronchopulmonary dysplasia (3), subglottic stenosis (1), acute transverse myelitis (1), patent ductus arteriosus (2), tracheomalacia (1)	3–14; *M* = 10.2										
Link et al. [11]	30	37	Motor vehicle crash (8), neurological disorders (7), gunshot wound or stabbing (3), laryngotracheal abnormalities (3), acetaminophen overdose (1), bronchopulmonary dysplasia (1), genetic syndrome (3), patent ductus arteriosus (1), gastroenterologic (2), poor feeding (1)	0.36–240; *M* = 124.8										
Marques et al. [15]	100	30	Neurologic disorder (e.g., hypotonia, cerebral palsy, stroke, asphyxia) (33), gastroesophageal reflux (25), history of rec. pneumonia (26)	1–288; *Mdn* = 32.7										
Mills et al. [16]	11	NR	Isolated Pierre-Robin sequence (100%)	*M* = 1.1 ± 0.6										
Pavithran et al. [24]	23	52	Laryngomalacia: without comorbidity (12), neurologic diagnosis and/or low muscle tone (5), Down Syndrome (2), primary congenital cardiac anomalies (4), premature born (4), repaired tracheoesophageal fistula (2)	0.23–8.5										
Richter et al. [25]	65	43	Neurological disorders (80%), congenital cardiac disease (31%), genetic syndrome (26%), gastroesophageal reflux disease (23%), prematurity (23%), upper aerodigestive tract anomalies (20%), seizure disorder (57%)	0.4–36; *M* = 9.9 ± 9.8										
Sitton et al. [29]	50	NR	Laryngomalacia/supraglottoplasty: Isolated (5), gastroesophageal reflux disease (48), neurologic disease (11), cardiac disease (18), genetic disorder (17)	0.3–26.8; *Mdn* = 4.5										
Suiter et al. [30]	79	44%	Neurologic disorder (25), genetic disorder (28), congenital heart defect (22), prematurity (15), vocal fold dysfunction (17), micrognathia (3), tonsillar hypertrophy (11)	0.4–170; *M* = 30 ± 26.4										
Suskind et al. [17]	56	41	Surgery (13), general medical (8), pulmonary (2), cancer (2), stroke (3), TBI (10), progressive neurological (7), cervical spinal cord injury (3), acute encephalopathy (3), seizure disorder (1), other neurological (4)	24–216; *M* = 160.8 ± 56.4										
Suterwala et al. [18]	17	25*	Gastroesophageal reflux disease	1–9.7; *M* = 5.4 ± 3.7										
Ulualp et al. [31]	25	60	Respiratory distress syndrome, NICU	8.5–11.3; *M* = 9.2 ± 0.7										
Umay et al. [32]	40	30	Gastroesophageal reflux (17), asthma (8), seizure disorder (15), cerebral palsy (5), Down syndrome (1), velocardiofacial syndrome (3), neonatal hypotonia (2), excision of posterior fossa ependymoma (1), spinal muscular atrophy (1), trisomy 9 (1), no comorbidity (3)	3–204										
Vetter-Laracy et al. [19]	251	31,5	Cerebral palsy (100%)	48–96; *M* = 68.4 ± 20.4										
Willette et al. [20]	62	34	Premature born	8.7–10.1 (PMA); *Mdn* = 9.1										
Armstrong et al. [12]	23	39	Neurologic (10), structural (6), cardiorespiratory (3), normal (4)	0.4–10; *M* = 3.2										

^a^Age at first enrollment, ^b^ multiple diagnosis possible, ^c^*Mdn* median, *M* mean and *SD* standard deviation

*From a sample of 28

The overall gender distribution could be calculated for a sample of 18 studies. Among 855 children, an average of 39% were girls. Four studies [13, 15, 25, 28] including Hartnick et al. with the biggest sample size did not report on gender, so the distribution remains unclear for the remaining 682 children.

Further analysis of the samples showed that three studies included only infants from the neonatal intensive care unit (NICU) [12, 14, 18], one focused on mechanically ventilated children [23], and two had a surgical focus [13, 25]. Six studies related to a single diagnosis or symptom: Congenital Zika Syndrome [22], isolated Pierre-Robin sequence [15], laryngomalacia [16] and gastroesophageal reflux disease (GERD) [17], cerebral palsy [26], and prematurity [19]. The remaining 10 samples varied widely in terms of principal diagnosis [10, 11, 20, 21, 24, 27–31] (Table 4).

### Implementation Protocols

FEES examinations were usually performed by a (pediatric) otolaryngologist and attended by one or two speech-language pathologists (SLP) or occupational therapists (OT) and a nurse. Two studies reported the performance of FEES by an SLP [12, 18], one by a pediatric neurologist [27], and one by a pediatrician [19]. Five authors did not provide information on the specialization of the person performing FEES [13, 15, 17, 25, 31] (Table 6).

The positioning of the children during endoscope insertion was most frequently reported as *upright*, as *upright as possible*, or *semi-reclined* with stabilization of the head. When breastfed infants were examined, the position preferred by the mothers was adopted after insertion of the endoscope. Five protocols did not include information on the positioning of the child [13, 22, 23, 29, 30].

In some cases, it was explicitly stated that feeding tubes were removed [12, 18] or not removed [16, 23]. Eighteen study protocols did not report on that topic.

Three studies exclusively focused on breastfeeding [12, 16, 20] and seven on bottle-feeding [13–15, 18, 19, 23, 25]. Two even provided standardized information on the type of nipple and consistency of milk [14, 18]. Five studies indicated that the boluses evaluated were *developmentally appropriate* [11, 17, 28, 29, 31]. Standardized boluses were reported in seven protocols, four of which specified the type [21, 24, 27, 32] and three the type and size of the boluses [10, 22, 30] (Table 6).

### Equipment

Most examinations were performed with a fiberoptic rhino-laryngoscope. In one study each, a video rhino-laryngoscope and a video bronchoscope were used. The diameters of the endoscopes ranged from 1.9 to 4.1 mm. In two studies, sensory testing via an air impulse channel (FEESST) was also included [25, 31]. Four studies did not report in detail on the endoscope used (Table 6).

In 10 protocols, topical anesthesia was administered as standard. In most cases, lidocaine gel was applied directly to the endoscope. Nine authors reported not using topical anesthesia, and three did not specify. The use of nasal decongestant was reported in two protocols [27, 28].

To calm infants during the uncomfortable insertion of the endoscope, calming techniques were employed in four studies using sucrose solution, non-nutritive sucking or breastfeeding [12, 14, 18, 20].

The use of a thickener was not applicable in the breastfeeding protocols. It was reported for eight studies, four of which indicated the type of thickener as modified corn starch [15, 19, 21, 22] and two as rice cereal [18, 24] (Table 6).

Food dye was reported in fourteen protocols (seven used green, five blue, one yellow in addition to blue, and two did not specify which color). Six studies did not report if they used a food dye and two did not use color but standardized yellow pudding and milk [30] or just white milk [23] (Table 6).

### FEES Procedural Steps

The assessment of anatomic structures was included in 14 protocols by mentioning *pharyngeal and laryngeal anatomical structures* without going into detail. Two of these also named the nasal airway, soft palate, and oropharynx [16, 20]. Ten protocols involving evaluation of secretion management, five protocols testing laryngeal sensitivity at that particular time point [17, 24, 25, 28, 31], and one assessing it as the final step of FEES [11]. In total, sensory testing was performed in six protocols, in one study using the touch method [24] and five using an air pulse [11, 17, 25, 28, 31]. Direct swallowing assessment was the core of all protocols with a single exception. Leder & Karas [10] referred to the standard protocol of Langmore [4] and did not provide further details. Compensatory strategies such as re-positioning, modification of texture, or pacing were part of seven protocols [13, 14, 19, 20, 22, 28] (Table 5).

**Table 5:** FEES implementation

Study	FEES performed by; assistance	Positioning of the child/adolescent	FEES procedural steps
Armstrong et al. [12]	*SLP*; OT, lactation consultant nurse, otolaryngologist, neonatologist	With endoscope in place: mother’s preferred position	2. Direct assessment of swallowing (not reported in detail)
Averin et al. [13]	*NR;* NR	NR	1. Observation of anatomical structures and a) secretion management 2. Direct assessment of swallowing 3. Compensatory strategies
Beer et al. [27]	*Pediatric neurologist;* two SLPs, nurse	Individually: buggy, wheelchair, nurse’s lap, bed	1. Observation of anatomical structures and a) secretion management 2. Direct assessment of swallowing
da Silva et al. [21]	*Otolaryngologist;* SLP	Sitting	2. Direct assessment of swallowing (not reported in detail)
Hartnick et al. [28]	*Otolaryngologist;* SLP, nurse	On caretaker´s lap or accompanied; stabilized while telescope is introduced	1. Observation of anatomical structures and a) secretion management and b) sensory testing (air pulse) 2. Direct assessment of swallowing 3. Compensatory strategies
Kamity et al. [14]	*Pediatric otolaryngologist; *neonatologist, SLP, nurse	Tightly swaddled, semi-reclined position at 45–90° angle, feeder stabilizes head	1. Observation of anatomical structures 2. Direct assessment of swallowing 3. Compensatory strategies (if necessary)
Leal et al. [22]	*Otolaryngologist;* SLP	NR	Reference to standard FEES protocol (Langmore [4]): 1. Observation of anatomical structures 2. Direct assessment of swallowing 3. Compensatory strategies (if necessary)
Leder et al. [23]	*Otolaryngologist;* NR	NR	Reference to standard FEES protocol (Langmore [4]) with slight modification: 1. Observation of anatomical structures 2. Direct assessment of swallowing (evaluation of the first 6–20 boluses)
Leder & Karas [10]	*Otolaryngologist;* NR	Upright	Reference to standard FEES protocol (Langmore [4]), no further specification
Link et al. [11]	*Pediatric otolaryngologist;* SLP	Upright, sitting on the lap, head stabilized	1. Observation of anatomical structures and a) secretion management 2. Direct assessment of swallowing 3. Sensory testing (air pulse)
Marques et al. [15]	*NR;* NR	On mother’s lap	2. Direct assessment of swallowing (not reported in detail)
Mills et al. [16]	*Pediatric otolaryngologist;* SLP, nurse, lactation consultant	On mother’s lap, latching after insertion of endoscope	1. Observation of anatomical structures and a) secretion management [Securing endoscope with rubber band and latch] 2. Direct assessment of swallowing 3. Compensatory strategies (re-positioning)
Pavithran et al. [24]	*Otolaryngologist;* SLP	45–90° reclining position on caretaker’s arm	1. Observation of anatomical structures and a) secretion management and b) sensory testing (touch method) 2. Direct assessment of swallowing (in case of aspiration, repetition of the consistency)
Richter et al. [25]	*NR;* NR	Upright or semi-reclined at caregiver’s lap with gentle restraint	b) Sensory testing (in 28 children, air pulse) 2. Direct assessment of swallowing
Sitton et al. [29]	*Otolaryngologist;* SLP, nurse	NR, stabilization of head by caregiver or nurse	1. Observation of anatomical structures and a) Secretion management 2. Direct assessment of swallowing
Suiter et al. [30]	*Otolaryngologist;* NR	NR	Reference to standard FEES protocol (Langmore [4]) with slight modifications: 1. Observation of anatomical structures 2. Direct assessment of swallowing
Suskind et al. [17]	*NR;* NR	Upright or semi-reclined at caregiver’s lap with gentle restraint	b) Sensory testing (air pulse) 2. Direct assessment of swallowing
Suterwala et al. [18]	*SLP;* OT	Swaddled, placed in the feeder’s arms in an elevated side-lying position at 20–30° elevation	1. Observation of anatomical structures and a) Secretion management 2. Direct assessment of swallowing
Ulualp et al. [31]	*NR;* NR	On the caregiver’s lap, upright	b) Sensory testing (air pulse) 2. Direct assessment of swallowing
Umay et al. [32]	*Otolaryngologist;* NR	Highest possible upright sitting position	2. Direct assessment of swallowing (not reported in detail)
Vetter-Laracy et al. [19]	*Pediatrician;* nurse	On the caregiver’s lap, stabilized head during the procedure	1. Observation of anatomical structures and a) Secretion management 2. Direct assessment of swallowing 3. Compensatory strategies
Willette et al. [20]	*Otolaryngologist;* two SLPs, nurse	Nurse stabilizes head while insertion; breastfeeding in position typically used	1. Observation of anatomical structures and a) Secretion management 2. Direct assessment of swallowing 3. Compensatory strategies (in case of unsafe breastfeeding)

*SLP* speech-language pathologist; *OT* occupational therapist; *NR* not reported

**Table 6:** FEES equipment

Study	Type (designation), manufacturer, diameter (mm)	Topical anesthesia and decongestion, application	Calming techniques	Volume and consistencies	Thickener	Food dye and dosage
Armstrong et al. [12]	Fiberoptic (ENF-XP), Olympus, 2.2	None	0.2 ml sucrose solution (pacifier), calming strategies	Breastfeeding	NA	Green food dye McCormick (Sparks, Maryland), 0.05 ml in 15 ml expressed human milk via syringe prior to latch
Averin et al. [13]	NR, NR, 2.2	Lidocaine gel (2%), on endoscope	NR	Breast milk or formula (small tastes)	NR	NR
Beer et al. [27]	Video bronchoscope (BF-3C160), Olympus, 3.8	Decongestive nasal drops, no topical anesthesia	NR	a. Fruit puree b. Liquid c. Bread (different consistencies dependent on aspiration risk)	NR	Colored in blue
da Silva et al. [21]	Fiberoptic, Machida Endoscope, 3.2	NR	NR	a. Liquid (apple juice) b. Puree (1/2 measuring spoon thickener, apple juice powder, and 100 ml water)	Modified instant corn starch (Nutilis, Support, Sao Paulo, Brazil)	Liquid yolk-colored food dye liquid indigo blue (Mix, Sao Paulo, Brazil)
Hartnick et al. [28]	Fiberoptic, NR, NR	1:1 tetracaine/oxymetazoline hydrochloride	NR	Developmentally appropriate	NR	Food coloring
Kamity et al. [14]	Fiberoptic, Pentax, 2.4	None	Sucrose solution (24%, pacifier)	Thin barium (50% dilution), 30 ml (similac volu feeder)	NR	Green Food Color McCormick (Sparks, MD, USA), two drops
Leal et al. [22]	Fiberoptic, Machida Endoscope, 3.2	NR	NR	a. Liquid b. Thickened liquid (50 ml/3 g) 1 ml, 3 ml, 5 ml via syringe c. Foodpaste or puree (2 spoons)	Modified corn starch (Sustap, Prolev, Brazil)	Liquid indigo blue food dye
Leder et al. [23]	Fiberoptic (ENF-P3), Olympus, 3.6	None	NR	Liquid (milk/formula) via bottle	NR	None (white milk)
Leder & Karas [10]	Fiberoptic (ENF-XP or ENF-P3), Olympus, 2.2 or 3.6	None	NR	Bottle-fed: clear or nectar-thickened liquid; others: a. Puree (custard, 5 ml) b. Liquid (milk, 5 ml) c. Solid (i.e., cracker, if indicated)	Yes/NR	Blue dye
Link et al. [11]	Fiberoptic (FNL 10 AP), Pentax, 3.2	Topical nasal anesthetic	NR	a. Liquids b. Variety of developmentally appropriate textures	NR	Green food color
Marques et al. [15]	Fiberoptic (ENF-P4), Olympus, 3.2	None	NR	Liquid or thickened liquid (milk) through bottle	Modified corn-based flour	Aniline color (blue)
Mills et al. [16]	Fiberoptic, Telepack Storz, 1.9	Lidocaine gel (2%), on endoscope	NR	Breastfeeding	NA	NR
Pavithran et al. [24]	Videoscope (11,101 VPS), Telepack Storz, 3.7	Xylometazoline, Lidocaine gel (2%), on endoscope	NR	Developmentally appropriate a. Thin b. Thick c. Puree	Rice cereal	Apple green dye
Richter et al. [25]	Fiberoptic, or fiberoptic and air pulse channel (FNL 10 AP), KayPENTAX, 2.5 or 4.0	Lidocaine gel (2%), outer surface of endoscope	NR	Formula Bottle-fed	NR	NR
Sitton et al. [29]	NR, NR, NR	None	NR	Developmentally appropriate	NR	Standard household green food coloring, one drop per 4–8 oz
Suiter et al. [30]	Fiberoptic (ENF-P3), Olympus, 3.6	None	NR	a. Puree (pudding, 3 × 5 ml) b. Liquid (milk, 3 × 5 ml)	NR	None (yellow puree and white milk)
Suskind et al. [17]	Fiberoptic (FNL 10 AP), Pentax, 4.0	Lidocaine gel (4%), outer surface of endoscope	NR	a. Liquids b. Variety of developmentally appropriate textures	NR	NR
Suterwala et al. [18]	Fiberoptic (ENF-XP), Olympus, 2.2	None	2 ml Sucrose solution (24%), NNS, sound and light reduction	a. Thin consistency (breastmilk or formula via slow-flow nipple) b. Subsequent consistencies and nipple types based on response to initial bottle	Rice cereal (Beech-Nut, USA (breastmilk not thickened)	Green food dye McCormick (Sparks, MD, USA)/, two drops 0.1 ml/30 ml bottle
Ulualp et al. [31]	Fiberoptic and air pulse channel (ENT-1000), Vision Sciences, 2.4	Lidocaine gel (2%), cotton-tipped applicator	NR	a. Liquids b. Variety of developmentally appropriate textures	NR	NR
Umay et al. [32]	Fiberoptic, Storz, 3.4	NR	NR	a. Liquid (water) b. Semi-solid (thickened water) c. Solid (bread)	Yes/NR	NR
Vetter-Laracy et al. [19]	Fiberbronchoscope (BF-XP 190), Olympus, 3.1	Lidocaine (2.5%) and prilocaine (2.5%) gel (Emla creme)	NR	a. Liquid (formula bottle-fed) b. Thickened formula (in case of aspiration)	Modified corn starch (Resource Thickenup), 6.4 g/100 ml	Dye, one drop
Willette et al. [20]	NR, NR, 2.7	Lidocaine gel (4%), distal end of endoscope	Breastfeeding	Breastfeeding	NA	Standard green food coloring via toothette (oral care stick)

*NA* not applicable, *NR* not reported, *NNS* non-nutritive sucking

**Table 7:** FEES outcome

Study, country	Design	FEES-based outcome (result/sample size) (in case of repeated testing, first measurement)	Complications
Armstrong et al.[12] USA	Prospective, cross-sectional, pilot	Penetration of milk (1/2), secretion (1/2); aspiration of milk (0/2), secretion (1/2)	None
Averin et al. [13] USA	Retrospective, cross-sectional	Swallowing dysfunction (10/63)	None
Beer et al. [27] Germany	Retrospective, cross-sectional	Penetration: Saliva (5/30), puree (7/24), thin liquid (5/21) Aspiration: Saliva (10/30), puree (7/24), thin liquid (7/21) Silent aspiration: Saliva (9/30), puree (3/24), thin liquid (1/21)	Short dips of oxygenation (< 85%, *n* = *2),* spontaneous recovery
da Silva et al. [21] Brazil	Prospective, cross-sectional	(Observer 1 and observer 2/N) Early spillover: Puree (5 and 8/30), liquid (9 and 14/30) Pharyngeal residue: Puree (13 and 9/30), liquid (10 and 9/30) Penetration: Puree (4 and 4/30), liquid (15 and 13/30) Aspiration: Puree (0 and 0/30), liquid (6 and 4/30)	NR
Hartnick et al. [28] USA	Retrospective, cross-sectional	Diagnostic categories according to Burklow et al. (1998) baseline feeding recommendations	NR
Kamity et al. [14] USA	Prospective, cross-sectional, pilot	Penetration (5/5), aspiration (3/5)	None
Leal et al. [22] Brazil	Retrospective, case series	Premature spillage (9/9), delay swallowing reflex (8/9), hypopharyngeal residue (4/9), PAS 1 (1/9), PAS 5 (3/9), PAS 7 (4/9), PAS 8 (1/9)	None
Leder et al. [23] USA	Prospective, cross-sectional	Aspiration or unsafe swallow (1/5)	NR
Leder & Karas [10] USA	Prospective, cross-sectional	Not systematically reported; no findings (13/23), aspiration (5/10; silent: 3), aspiration (3/7)	NR
Link et al. [11] USA	Retrospective, cross-sectional	Hypopharyngeal secretion: None (48/100), minimal (21/100), moderate (9/100), severe (22/100), penetration (46/96) aspiration (31/96) LAR absent (22/100)	NR
Marques et al. [15] Brazil	Prospective, observational	Aspiration risk (7/11) defined as milk reflux, delayed initiation of swallowing or residue	NR
Mills et al. [16] New Zealand	Retrospective, cross-sectional	Aspiration and/or penetration (15/23), silent aspiration (10/23)	None
Pavithran et al. [24] India	Prospective, cross-sectional	Glottic secretion (17/65), excessive pharyngeal secretion (23/65), premature spillage (44/65), pharyngeal residue (33/65), penetration (42/65), aspiration (15/65), weak/absent LAR (16/65)	NR
Richter et al. [25] USA	Retrospective	Penetration (44/50), aspiration (36/50), LPST (mm Hg in 28 patients: *M* = 8.23 ± 1.85)	NR
Sitton et al. [29] USA	Retrospective, cross-sectional	Report on feeding recommendations, spillage, penetration, aspiration, and residue included in logistic regression	NR
Suiter et al. [30] USA	Prospective, cross-sectional	Aspiration included in test statistics for reference test	NR
Suskind et al. [17] USA	Retrospective, cross-sectional	Hypopharyngeal pooling (15/17), LPST (mm Hg *M* = 6.3 ± 1.0, penetration (5/17), aspiration (7/17)	NR
Suterwala et al. [18] USA	Prospective, cross-sectional	Penetration and aspiration included in intra- and interrater-reliability	None
Ulualp et al. [31] USA	Retrospective, cross-sectional	Laryngopharyngeal sensation: Normal (6/40), moderate (20/40), severe (10/40), no response (4/40) pharyngeal pooling (24/40), premature spillage (17/40), residue (6/40), penetration (14/40), aspiration (10/40)	NR
Umay et al. [32] Turkey	Prospective, cross-sectional	Dysphagia level according to Warnecke et al. [43], self-developed classification: 1 = normal (29/251), 2–3 = mild (72/251), 4–5 = moderate (79/251), 6 = severe (71/251)	NR
Vetter-Laracy et al. [19] Spain	Retrospective, cross-sectional	Pharyngeal pooling (14/62), penetration/aspiration (44/62), signs of GERD (17/62), residue (24/62)	None
Willette et al. [20] USA	Retrospective, cross-sectional, case series	Functional swallowing (2/24), penetration (20/24), aspiration (12/24)	None

*NR* not reported, *PAS* penetration-aspiration scale, *LAR* laryngeal adductor reflex, *LPST* laryngopharyngeal *sensory* threshold, *GERD* gastroesophageal reflux disease

### FEES-based Outcome

In some cases, there was a discrepancy between the FEES-based result advertised in the method and the actual outcome presented in the results. The following section refers to the actual parameters reported in the results.

The parameter combination of secretion pooling and laryngeal sensation was reported in four studies [11, 17, 24, 31], one study reported only secretion pooling [19]. Premature spillage was stated as present or absent in four [21, 22, 24, 31] and delay of swallowing reflex in one study [22]. Penetration was reported in 11, aspiration in 13, and silent aspiration in four studies [10, 16, 22, 27]. Two studies summarized penetration-aspiration [16, 19]. Residues were reported in five result sections [19, 21, 22, 24, 31]. Only one study [22] used Rosenbek’s penetration-aspiration scale (PAS) [33]. No validated scale was used for any other outcome parameter (Table 7).

Thirteen of the studies lacked any information regarding complications or adverse events; in the remaining studies, none were reported.

### Subgroup Protocols

FEES protocols for sub-groups could be identified for exclusively breastfed and bottle-fed infants. The breastfed subgroup consisted of 51 participants from three studies (43.5% female) aged 0–10 months [12, 16, 20] (Table 4). An otolaryngologist or an SLP performed the endoscopy. At least one SLP or OT and a nurse assisted. The diameter of the endoscope ranged from 1.9 to 2.7 mm. Lidocaine gel as topical anesthesia was put onto the endoscope according to two protocols [16, 20]. Sucrose solution or breastfeeding in advance was used as a calming strategy in two studies [12, 20]. In two protocols, food dye was applied prior to latching via oral care swab or syringe (Table 6). Mills et al. [16] secured the endoscope with a rubber band before latching. After insertion of the endoscope, all children were positioned in their preferred breastfeeding position. One protocol described only *direct assessment of swallowing* [12], while the two other studies included the stages *observation of anatomical structures and secretion management*, *direct assessment of swallowing*, and *compensatory strategies* (Table 5). *Penetration* and *aspiration* of milk, considered separately, were the endpoints reported in two studies. The third study summarized *penetration-aspiration* (including *silent aspiration*) without reporting each item individually [16] (Table 7).

The bottle-fed subgroup was based on seven studies and consisted of 221 children aged 0–26 months [13–15, 18, 19, 23, 25]. The gender was reported for 97 children of whom 38.5% were female (Table 4). FEES was performed by an otolaryngologist, an SLP, or a pediatrician, usually assisted by an SLP or OT. Three of the seven studies did not include information on the examiner's profession. The diameter of the endoscope ranged from 2.2–3.6 mm, in case of additional sensory testing via air pulse 4 mm [25]. Mostly, the position of the child was semi-reclined. Three protocols reported on the use of topical anesthesia [13, 19, 25] and two reported on standardized volumes and consistencies [14, 18], whereas the protocol by Kamity et al. [14] included barium due to the simultaneous videofluoroscopy. Five studies used food coloring, three used thickening agents (Table 6). *Assessment of anatomic structures, direct evaluation of swallowing*, and *compensatory strategies* was included in three protocols, two of which also included *secretion management*. [13, 19]. In three protocols, the individual steps of the implementation were not described in detail (Table 5). *Aspiration* was reported in three studies [14, 23, 25]. *Penetration alone* was specified in two [14, 25]. One study reported on *swallowing dysfunction* [13]. One study reported on *aspiration risk* [15], one summarized *penetration-aspiration* [19], and one study did not report an exact number of penetration and aspiration in the sample but included it in intra-and interrater correlation [18] (Table 7).

## Discussion

The aim of this review was to identify implementation protocols for pediatric FEES described in research studies and to analyze those in detail in terms of procedural steps, equipment, and reported outcomes. It provides important insights into the critical lack of standardization in pediatric FEES protocols and FEES-based studies. It also reflects a rather poor methodological quality of the studies. For this reason, conclusions are limited.

### Sample

A wide variation in age, diagnoses, and health conditions of the children evaluated was found both between included studies but also within them. Interestingly, girls accounted for only 39% of the total population (with some missing data). The interesting question here is whether dysphagia in children is more common in one gender. It would therefore be important to also report a gender-differentiated outcome.

### Protocols

All implementation protocols were described incompletely and differed in many aspects. As no validated pediatric FEES protocols exist to date, no study could be based on such a protocol. The first comprehensive pediatric protocols were published in 2020 [1, 3], so future studies will be expected to increasingly refer to these. No uniform recommendations for equipment to be used in pediatric FEES have been published to date. A key point to consider here is that very thin, modern chip-on-tip videoscopes are likely to give the best results, while fiberoptic endoscopes allow cost-effective area-wide use. Obtaining full details of the diameter and type of endoscope and other equipment, FEES team, nasal decongestant, topical anesthesia, calming techniques, positioning of the child, and thickening and dyeing of the bolus would enable a better comparison of the examination and the outcome. However, the data from the investigated studies do not allow for comparison.

In summary, the procedural steps proposed by Miller et al. [3] can be reproduced in most protocols. The *direct assessment of swallowing* is included in all protocols. However, the further descriptions of the study protocols are not detailed enough to allow replication and evaluation.

Few study protocols included standardized bolus amounts and consistencies commonly found in adult protocols. Although children's eating behaviors are distinctly individual, simply stating "developmentally appropriate" is not sufficient for study protocols and should be appropriately specified.

For the reported outcome a similar picture as for procedural steps became apparent. Two problems can be identified here: (i) there are no valid outcome measurement scales for pediatric FEES (ii) the recording of FEES outcome was insufficient for retrospective studies. In a retrospective analysis of pediatric FEES data obtained in our hospital [34], we demonstrated that a large number of missing values were due to incomplete documentation and lack of standardization of protocols.

Since the high rate of silent aspiration in pediatric samples is repeatedly pointed out [35, 36], it would have been very interesting to investigate the factor of silent aspiration for the complete sample. Unfortunately, only four of the 22 studies reported silent aspiration as an outcome parameter.

Overall, no adverse events occurred and FEES was considered safe in all groups, consistent with the findings of Miller and Willging's 25-year experience [1]. However, not all studies consistently reported complications or how many examinations were discontinued or could not be performed at all. This issue is particularly evident in retrospective studies, primarily including cases with a complete FEES and not systematically recording how many FEES could not be performed.

The establishment of specific protocols for breastfed and bottle-fed infants is advisable. Future protocols should take into account that many children, though still breastfed additionally eat puree or are bottle-fed and already receive solid foods.

### Limitations

Based on the recently published systematic review by Dharmarathna et al. on quantitative instrumental studies of swallowing in children [6], the methodological quality of pediatric FEES studies was expected to be poor, and the inclusion criteria were expanded accordingly. Meta-analysis of the data was not possible because of a large number of missing data and the range of outcome parameters. In particular, the retrospective studies with large samples had significant deficiencies in the sample description and specification of the data, making further analyses and comparisons impossible. In principle, retrospective analyses of patient data are valuable if they meet certain requirements and systematically provide the necessary data.

### Implication for Practice

In practice, the implementation and documentation of pediatric FEES should be standardized and adapted specifically for children and adolescents. Depending on age and nutritional status, fixed procedures and evaluation forms should be available. Since patient groups in practice tend to be heterogeneous, a modular approach may be useful. For infants and young children, a small endoscope, calming techniques, and, where appropriate, a thickening agent and a food dye that can be used safely for infants should be available. For therapy planning and diagnostics, but also to gain more experience, the parameters *secretion management and pharyngeal secretion pooling*, *premature spillage*, *delay in swallowing reflex*, *penetration, aspiration (and clearing)*, *silent aspiration*, *residue,* and *laryngeal sensation* should also be recorded and documented in practice. This is already standard in adults or suggested in recently published recommendations [1, 3].

### Implication for Future Research

As a general implication retrospective and prospective studies should focus more on specific age or diagnosis groups. For rare diseases, case numbers should be increased through multicenter collaboration or meta-analysis. For this purpose, FEES protocols must be described in sufficient detail to allow replication. This includes the FEES performing team, technical and other equipment, bolus types and sizes, calming strategies, exact procedural steps, and outcome.

The *positioning* of the child during insertion of the endoscope and throughout the subsequent examination, as well as the entire setting, should be described and illustrated with a photograph or drawing. *Calming techniques* such as sucrose solution and non-nutritive sucking, distraction by videos, or consultation with a child life specialist (as suggested by Miller & Willging [1]) should be mentioned.

A systematic report of the outcome is essential. Based on valid scales for adult FEES, the evaluation of all parameters should be recorded in scale form rather than just as present or absent. However, adaptation and validation of those scales for pediatric FEES are still needed: *pharyngeal secretion pooling* (e.g., Murray secretion scale [37]), *premature spillage* (e.g., Langmore and colleagues [38]), *delayed swallowing reflex* (e.g., Warnecke and colleagues [39]) *penetration (alone), aspiration and clearing, silent aspiration* (e.g., PAS [33]), *residue* (e.g., Yale Pharyngeal Residue Severity Rating Scale [40]), and *laryngeal sensation* (e.g., Marian et al. [41]). Preferably, results are also reported for each gender separately. Findings of interest, specific to certain groups should also be reported.

A final important issue for future research concerns compliance and general behavior of children during FEES. Future studies should report whether excessive crying, severe resistance, or refusal to eat or drink occurred during the examination and how this affected the acquisition of meaningful swallowing images. By specifying the average duration of the examination and after what time and how it was possible to calm down the child or not, it would help in future to find out more about the acceptance of the examination (e.g., in certain age groups). In addition, it should be summarized how many examinations had to be prematurely terminated or could not be performed at all. Of course, other reasons for termination of examinations such as choanal stenosis should also be given.

Researchers and practitioners using FEES should always keep in mind that swallowing function can be distorted by strong, sustained crying or discomfort. The starting point for a meaningful study should therefore always be the greatest possible comfort for the children and their parents. Future research must deal with how this comfort can be achieved.

## Conclusion

There is currently no pediatric FEES protocol that fully addresses the implementation, equipment, and, most importantly, outcome. Promising approaches are offered by protocols for infants who are breastfed, bottle-fed, or cared for in the neonatal intensive care unit. Even though the included studies did not exhibit good methodological quality and lack of data did not allow for direct comparison, this systematic review provides an important foundation for future pediatric FEES studies. An invaluable basis for this is provided by the empirical values and innovative ideas of the authors and researchers of the included studies.
